# Salivary nitrite content, cognition and power in Mixed Martial Arts fighters after rapid weight loss: a case study

**Published:** 2016-06-19

**Authors:** Nathalia F. Camarço, Ivo V. Sousa Neto, Dahan C. Nascimento, Jeeser A. Almeida, Denis C. L. Vieira, Thiago S. Rosa, Guilherme B. Pereira, Jonato Prestes

**Affiliations:** 1 *Department of Physical Education, Graduation Program on Physical Education, Catholic University of Brasilia, Brasília, Brazil*; 2 *Department of Physical Education, University Center of Federal District, Brasília, Brazil*; 3 *Department of Physical Education, Federal University of Mato Grosso do Sul, Campo Grande, Brazil*; 4 *Graduation Program on Biotechnology, Dom Bosco Catholic University, Mato Grosso do Sul, Campo Grande, Brazil*

**Keywords:** dehydration, exercise performance, athletes, combat sports

## Abstract

**Background and Aim:**

Rapid weight loss (RWL) is extensively practiced by combat sports athletes, including Mixed Martial Arts (MMA), but its effects on performance are not well established with different magnitudes of RWL, including those higher than 5% of total body weight. The aim of the present study was to follow MMA athletes during RWL with subsequent weight regain to evaluate the responses of isometric strength, power, cognition and salivary nitrite (${\text{NO}}_{2}^{-}$) content.

**Methods:**

Two professional male MMA fighters, same age, competing in the same weight category underwent two magnitudes of RWL before a simulated competition period. Anthropometric measures, records of nutritional status, training, voluntary dehydration strategies, salivary samples, cognition response, isometric strength and muscular power were obtained: (I) 7 days before combat, (II) at the weigh-in moment, and (III) in the combat day.

**Results and Conclusions:**

Athlete 1 lost 7.2 kg (9.1% of total bodyweight) and Athlete 2 lost 4.0 kg (5.3% of total bodyweight). Athlete 1 had a lower and misbalanced caloric ingestion (708 ± 428 kcal), ingested 6 L of water during the first 5 days of RWL, underwent 2 days of fasting, water and sodium restriction before weigh-in. Athlete 2 was supervised by a nutritionist, had a balanced diet (1600 ± 0 kcal), ingested 2 L of water during the first 6 days of RWL, underwent only 1 day of fasting and water restriction, and did not restrict sodium. As expected, there was a negative effect of RWL in the evaluated parameters at the weigh-in moment, while in the combat day, salivary ${\text{NO}}_{2}^{-}$) content.

**Methods:**

Two professional male MMA fighters, same age, competing in the same weight category underwent two magnitudes of RWL before a simulated competition period. Anthropometric measures, records of nutritional status, training, voluntary dehydration strategies, salivary samples, cognition response, isometric strength and muscular power were obtained: (I) 7 days before combat, (II) at the weigh-in moment, and (III) in the combat day.

**Results and Conclusions:**

Athlete 1 lost 7.2 kg (9.1% of total bodyweight) and Athlete 2 lost 4.0 kg (5.3% of total bodyweight). Athlete 1 had a lower and misbalanced caloric ingestion (708 ± 428 kcal), ingested 6 L of water during the first 5 days of RWL, underwent 2 days of fasting, water and sodium restriction before weigh-in. Athlete 2 was supervised by a nutritionist, had a balanced diet (1600 ± 0 kcal), ingested 2 L of water during the first 6 days of RWL, underwent only 1 day of fasting and water restriction, and did not restrict sodium. As expected, there was a negative effect of RWL in the evaluated parameters at the weigh-in moment, while in the combat day, salivary ${\text{NO}}_{2}^{-}$ was not completely reestablished at baseline levels (decreased by 35.9% in Athlete 1 and, 25.2% in Athlete 2, as compared with 7 days before). The athlete who underwent a lower weight loss (5.3%) presented better recovery of cognition and upper limbs power on the combat day as compared with the athlete who lost 9.1% of body weight. Although we cannot precisely conclude, this case report led us to believe that the recovery period between weigh-in and competition may be insufficient for total reestablishment of salivary ${\text{NO}}_{2}^{-}$ after RWL, and higher amounts of RWL

have negative impacts on average power and cognition when compared with lower RWL.

**Relevance for patients**: Scientific aspects related with performance in MMA athletes brought to light the absence of studies investigating the recovery of isometric strength, power, cognition and salivary ${\text{NO}}_{2}^{-}$ during RWL with subsequent weight regain. This study revealed that athletes from the same categories can adopt different magnitudes of weight loss, and that this procedure impacts several important measures, for example, the reduction of salivary ${\text{NO}}_{2}^{-}$ is associated with the lower O_2_ transport capacity, decreasing muscle performance.

## 1. Introduction

Athletes of combat sports have been using strategies of acute dehydration to promote rapid weight loss (RWL) and posterior regain of weight in a 12 – 36 hours interval between weigh-in and combat. These strategies are commonly seen in Judo, Brazilian Jiu Jitsu, Karate and Wrestling athletes [1]. This hypothetically led them to take some advantage of strength and power over an opponent who does not reduce weight. Generally, the major amount of weight is lost in the week preceding competition [2].

Dehydration of approximately 2% of body weight, can induce, impairment in performance, vigilance attention, short-term memory, perceptual discrimination, visuomotor tracking, and psychomotor skills [3,4,5] which can result in negative neurophysiological consequences that impair performance [6], including a decrease in salivary nitrite (${\text{NO}}_{2}^{-}$) concentration [7].

Salivary ${\text{NO}}_{2}^{-}$ concentration is a non-invasive marker of performance in athletes [8,9], since it is known to control muscular blood flow[10] and reduction of ${\text{NO}}_{2}^{-}$ is related with lower O_2_ transport capacity, decreasing muscle performance. A recent study by Yang et al. [7] showed a reduction of ${\text{NO}}_{2}^{-}$ content and nitric oxide (NO) production in taekwondo fighters who used the RWL strategy, evidencing negative effects in these athletes.

Furthermore, there is evidence that these strategies can trigger several changes in the health of athletes (eg. cardiovascular efficiency; hormonal imbalance and opportunistic infections), which in turn can interfere with physical performance.

Regarding the use of RWL and physical performance, data remains controversial [1]. The weight loss of approximately 5-6% of total bodyweight with a subsequent 4 h recovery period, did not affect strength performance in male athletes [11]. In this sense, RWL less than 6% of total body weight among athletes, seems to increase their likelihood of an untoward health event both before and during competition, due to a dehydration status [12], alter cognitive response [3,4,5], but not to impair muscular performance [11]. However, a very short period (< 1 h) of recovery result in negative effects on performance [13]. Thus, many MMA athletes have resorted to the use of RWL techniques, commonly used in other combat sports, taking advantage of the 24-36 h between weigh-in and the main event.

To the best of our knowledge, this is the first study to investigate weight loss and recovery, with the same interval of time that it occurs in a real MMA competition situation and different weight loss approaches. Moreover, we described nutritional and dehydration strategies during 8 days. None of the above mentioned studies investigated cognition in MMA athletes. Thus, the purpose of the present study was to describe the influence of RWL and weight regain on salivary nitrite concentration (indicative of NO concentration), power and cognitive indicators in two professional MMA fighters submitted to 7 days of RWL followed by a 36 h recovery.

## 2. Subjects and study design

### 2.1. Subjects

Two professional male MMA fighters competing in the lightweight class (145 to 155 lbs. or 65.7 to 70.3 Kg), with 3 years of professional MMA combat sport experience. The participants signed an informed consent document. This study was approved by the Local Research Ethics Committee for Human Use (protocol #763.992).

**Athlete 1**

2 wins, 1 loss, 0 draws; age: 22 years; height: 1.77 m; body mass: 79.0 kg; body mass index: 25.2 kg/m^2^;; body fat (BF): 9.0 % and without nutrition supervision. **Athlete 2**

4 wins, 2 losses, 0 draws; age: 22 years; height: 1.74 m; body mass: 75.5 kg; body mass index: 24.9 kg/m^2^;; BF: 7.6 % and with nutrition supervision.

### 2.2. Study design

We conducted the measures of cognition, isometric handgrip strength and power output at three moments: 7 days before competition (I), 36 h before competition (II), and competition day (III), in a simulated competition situation, simulating a pre-competition period, where athletes had 1 week to practice RWL before weigh-in and 1 recovery day before hypothetical competition.

Athletes trained, practiced RWL strategies and were on diet for 1 week before a competition simulation. The participants recorded their diet and training in a diary, and all food, training and dehydration strategies variables were evaluated throughout the duration of the study.

Athletes had three familiarization sessions for all tests data collection. All measurements took place at the same time (beginning 9 a.m.), seven days before simulated combat, 36 h before the combat day. Measurements were completed following overnight fasting, always in the same sequence: (1) nude bodyweight (Filizola, Brazil); (2) body composition, utilizing Pollock 7 skinfolds protocol [14]; (3) cognition, throughout the computerized version TESTINPACS^®^ [15] of the Stroop Color-Word Test (SCWT) divided into three tasks, as a mental stressor [16]; (4) hand grip, upper limbs power, and lower limbs power, described below. Diet, bodyweight and exercise were tracked daily and athletes were instructed to maintain nutritional habits during data collection. Athletes arrived in a fasting state. Following Jeacocke e Burke (2010) [17] recommendations, a solution containing 30 g of carbohydrate was provided. Athletes ingested this solution immediately after body composition assessment. Tests were performed thirty minutes after ingestion. Saliva samples were collected and stored at -80˚C from participants in all conditions [18,19].

One of the most important components of fighting during the competition is cognition and visual time reaction. In order to evaluate these aspects, the computerized version of the Stroop Color-Word Test (SCWT) divided into three tasks was used as a mental stressor [16]. In the first task, cues consisted of color-words (red, green, blue and black) and colored rectangles (neutral stimuli) presented in random order. The words were presented either in the color itself. The subjects were asked to indicate the color of the rectangle. In the second task, instead of rectangles, given words appear written in white color, cues are also words written in white color and subjects were supposed to choose the correspondent word by only reading. In the third task, words appear written in an incongruent color that the meaning (eg. the word “green” written in red ink) and subjects were asked to choose the cue with the right color of the word, without reading the actual word itself. Right and left keys presses were used as the mode of response. Time to response and number of mistakes are presented. The test was applied by a same evaluator at the same time, after standardized carbohydrate ingestion, always in a calm and clear room.

Due to the specificity of the test for MMA fighters and also due to possibility of reduced global strength from isometric handgrip [20], data on maximal handgrip strength were obtained using a JAMAR adjustable hand-held dynamometer (model BK-7498, Fred Sammons, Inc., Burr Ridge, IL). Three consecutive maximum attempts with 5 s duration and a 3 min rest (1min 30 s after each hand), were made and we used the best attempt. Subjects were verbal encouraged with previously standardized commands, by the same evaluator.

The power produced and sustained by upper limbs were measured by a linear position transducer (Peak Power, Cefise, Sao Paulo, Brazil) during chest press exercise on smith machine. Load was regulated at 30% 1RM (one repetition maximum), considering the optimal load do peak power production [21]. 1RM was tested in duplicate on the week that preceded tests.

For lower limbs, the capacity of power production and sustention was measured through 5 vertical jumps, interspersed by a 5 s rest between every two jumps on a contact platform (Cefise, São Paulo, Brazil). During both tests, subjects were verbal encouraged with previously standardized commands, by the same evaluator.

#### 2.2.1. Cognitive analyses

Computerized version of the Stroop Color-Word Test (SCWT) divided into three tasks was used as a mental stressor [16]. We considered the time to respond and the number of mistakes on the three tasks from Stroop Color-Word Test.

#### 2.2.2. Isometric handgrip strength analyses

We obtained relative values of isometric handgrip strength thought the equation: absolute handgrip strength (kg) / total bodyweight (kg) in each moment [20].

#### 2.2.3. Power analyses

Peak and average power (watts) produced during 5 repetitions in the concentric phases were determined by the manufacturer software (version 4.0; Peak Power software analysis) for upper limbs. Peak and average power (watts) were determined by the manufacturer software (Jump System Pro software analysis) for lower limbs [21]. The coefficient of variation (CV%) between repetitions was obtained thought the equation: CV = 100 (standard deviation / peak average in 5 repetitions).

#### 2.2.4. Salivary nitrite content analyses

Salivary nitrite concentration was measured by Greiss reaction [18] using a standard curve (0-80 µmol/L sodium nitrite) [19]. Nitrite is a widely used indicator of NO production.

## 3. Results

Calories from carbohydrate, protein, fat, water and dietary supplement consumption, RWL strategies and training reports are described in Table 1. There were differences in voluntary dehydration strategies and nutritional aspects. Athlete 1 had a lower and misbalanced caloric ingestion (708 ± 428 kcal), ingested 6 L of water during the first 5 days of RWL, underwent 2 days of fasting, water and sodium restriction preceding weigh-in. Athlete 2 was supervised by a professional nutritionist, had a balanced diet (1600 ± 0 kcal), ingested 2 L of water during the first 6 days of RWL, underwent only 1 day of fasting and water restriction, and did not restrict sodium.

Both athletes reported low intensities of training during the week. Volumes and days are described in Table 1. All procedures were descriptive, with no intervention on diet and training, preserving the usual habits practiced by athletes to achieve weight. Athlete 1 lost 7.2 kg, which corresponds to 9.1% of total bodyweight loss. Athlete 2 lost 4.0 kg, which corresponds to 5.3% of total bodyweight (Table 1).

**Figure 1.**
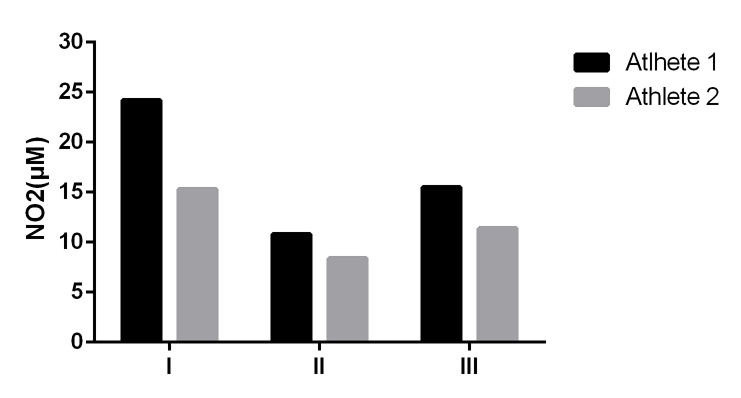
Values of nitrite (µM) at baseline (I), weigh-in (II) and combat day (III).

During the weighing period, athletes showed a reduction in salivary nitrite concentration (athlete 1: 55% and athlete 2: 45.1%). However, on the day of competition, through the replacement of body weight, the salivary nitrite concentration showed recovery when compared to dehydration period, but the baseline levels were not completely reestablished (athlete 1: 35.9% and athlete 2: 25.5% less than baseline values) (Figure 1).

Differences in cognition between moments, the response time to the cognitive tasks and the number of mistakes in each moment are presented in Table 2. Although we did not observe huge differences from the response time between athletes in each moment, the number of mistakes was higher in athlete 1 than in athlete 2, in all moments, and specifically increased on combat day.

Absolute and relative values of handgrip strength of athletes 1 and 2 are presented in Table 3. No notable differences were found between athletes in each moment for isometric strength for right and left hands.

**Figure 2.**
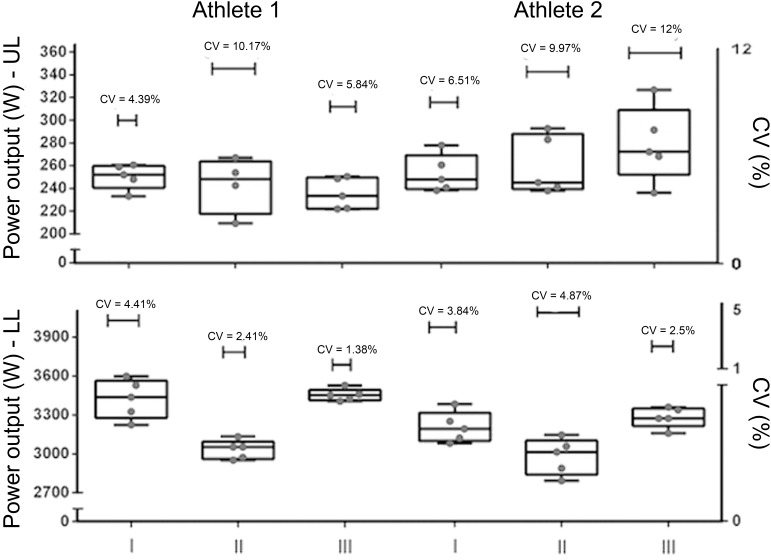
Upper Limbs (UL) and Lower Limbs (LL) power output and Coefficient of Variation (CV%) between repetitions at baseline (I), weigh-in (II) and combat day (III).

Upper (UL) and lower limbs (LL) average power, individual values for each repetition and coefficient of variation between repetitions in each moment is demonstrated in Figure 2. Muscular power output decreased in both athletes at the weigh-in day when compared with baseline values. Higher values for UL were recorded in the competition day for athlete 2 (5.3% loss) as compared with athlete 1 (9.1% loss).

**Table 1.** Training week report, diet and rapid weight loss strategies.

Athlete 1							Athlete 2						
Moment	Diet	Water	SR	Weight	ES	DS	Diet	Water	SR	Weight	ES	DS
Day 1 - Baseline	411/ 11-87-2	6 L	No	79	1 h whole body resisted training, 30 min boxing	No	1600/45-40-15	2 L	No	75.5	1 h 30 min fight technique, 30 min jogging	No
Day 2	1312/19-78-3	6 L	No	78	30 min jogging, 15 min jumping rope	No	1600/45-40-15	2 L	No	74.9	1 h hole body resisted training, 1 h 30 min fight technique	No
Day 3	512/31-67-1	6 L	Yes	77.7	No	No	1600/45-40-15	2 L	No	74.5	1 h 30 min fight technique, 30 min jogging	No
Day 4	997/14-82-4	6 L	Yes	76.8	1 h 30 min fight technique	No	1600/45-40-15	2 L	No	73.8	1 h whole body resisted training, 1 h 30 min fight technique	No
Day 5	310/66-44-0	6 L	Yes	76.5	30 min boxing	Exercise with plastic clothes	1600/ 45-40-15	2 L	No	73.7	1 h 30 min fight technique, 30 min jogging	No
Day 6	Fasting	Restriction	Yes	77.7	30 min jumping rope	Exercise with plastic clothes	1600/45-40-15	Restriction	No	73.8	1 h whole body resisted training, 1 h 30 min fight technique	1 h sauna
Day 7-Weigh in	Fasting	Restriction	Yes	71.8	No	No	Fasting	Restriction	No	71.5	No	1 h sauna
Day 8-Combat	2662/ 92-4-4	6L	No	77.9	No	No	2140/34-28-38	6 L	No	75.5	No	No

Diet = kcal/% carbohydrate – % protein – % fat. SR: sodium restriction; ES: exercise strategy; DS, dehydration strategy.

In addition, CV% of UL was increased on combat day in comparison with baseline in athlete 2. The same did not occur with athlete 1. Although CV% was increased in athlete 2, the minimum value of peak power on combat day, did not differ substantially of the minimum value of peak power on baseline Thereby, the increase in CV% between repetitions was due to increase in some repetitions performed by this athlete and not the opposite.

Interestingly, UL power output was reestablished with a 5.3% bodyweight loss, but not in a 9.1% of total bodyweight loss (athlete 1). In this sense, it seems that the magnitude of RWL can be a determinant factor for muscular power output recovery (Figure 2).

## 4. Discussion

Athlete 1 lost 7.2 kg (9.1% of total bodyweight) with a lower and misbalanced caloric intake (708 ± 428 kcal), the water ingestion was 6L during the first 5 days of RWL, underwent 2 days of fasting, water and sodium restriction preceding weigh-in. Athlete 2 lost 4.0 kg (5.3% of total bodyweight) with nutritional supervision, had a balanced diet (1600 ± 0 kcal), ingested 2L of water on the first 6 days of RWL, underwent only 1 day of fasting and water restriction, and did not restricted sodium. As expected, there were negative effects on evaluated parameters at the weigh-in moment, while in the combat day, salivary NO2 content was not completely reestablished as compared with baseline (decreased by 35.9% in Athlete 1, and 25.2% in Athlete 2, as compared with 7 days before). Athlete 2 (5.3% weight loss) presented better recovery of cognition and upper limbs power output on the combat day when compared with Athlete 1 (9.1% weight loss).

The amount of evidence on this issue with a follow-up of performance and cognition parameters considering diet and amount of weight loss in MMA athletes is very limited. The main results revealed that a seven-day RWL strategy negatively affected power output and cognition 36 h before competition. Additionally, the athlete who underwent a lower weight loss presented lower deficit in cognition and a better recovery of power output in the competition day when compared to the athlete who lost more weight.

The deleterious effects of RWL on the performance of athletes is well documented [22,23,24], while cognition was less studied in MMA athletes. Although comparisons between both athletes are hard to make, athlete 2 displayed a better cognition status in all moments. It has been shown that severe dehydration (>2% bodyweight loss) impairs cognition function in military subjects [25]. Some mechanisms, such as limited attention during a thirst situation and changes at total brain volume shrinkage and over-recruitment of specific brain areas [26,27] may partially explain the impaired cognitive function. A difficult issue is to compare results from other studies, because the protocols used to achieve RWL are widely variable, such as exercise, extreme environment, sweat suits and sever [28,29,30].

**Table 2.** Time to respond and number of mistakes during three tasks of the Stroop Color-Word Test.

Athlete 1				Athlete 2		
	I	II	III	I	II	III
Task 1	13.18 (±1.80)	14.89 (±1.86)	12.43 (±3.96)	14.32 (± 2.78)	11.90 (±2.94)	13.99 (±5.08)
Task 2	16.05 (± 1.27)	15.28 (± 5.72)	14.63 (± 3.96)	22.57 (± 15.1)	13.59 (±2.98)	16.01 (±1.74)
Task 3	20.82 (± 5.40)	21.68 (± 7.78)	20.78 (± 9.13)	29.44 (±15.15)	21.58 (±6.69)	20.53 (±5.62)
Mistakes	2	2	3	0	1	0

I= 7 days before competition; II= 36 h before competition; III= competition day; Data are presented as mean ± standard deviation (seconds) for each task and in absolute number for mistakes.

**Table 3.** Absolute and relative values of handgrip isometric strength.

Athlete 1				Athlete 2		
	7-d before competition	36-h before competition	Competition day	7-d before competition	36-h before competition	Competition day
Right handgrip	55.3 (0.7)	57.0 (0.7)	54.6 (0.7)	50.6 (0.7)	50.0 (0.7)	50.6 (0.6)
Left handgrip	56.0 (0.7)	56.6 (0.7)	52.6 (0.6)	44.6 (0.5)	47.0 (0.6)	48.6 (0.6)

The data are presented as absolute (kg) and in the parenthesis relative values. Relative values are described in absolute/ bodyweight (kg).

The strategies of RWL widely used in the combat sports field, especially in MMA, result in dehydration and negative effects may occur. Some negative effects include increase in heart rate and hematocrit in which may hinder O_2_ transport to the muscle cells [31]. It is understood that NO has several important functions in the organism including the control of muscle blood flow [10]. The reduction of NO is related to the lower O_2_ transport capacity, decreasing muscle performance. Furthermore, a recent study by Yang et al. [7] showed a reduction of nitrite content and NO production in taekwondo fighters who used the RWL strategy, evidencing negative effects of these athletes. Thereby, salivary nitrite content has been shown as an important non-invasive marker of performance in athletes [8,9]. Behavior is shown in Figure 2.

It is known that water and nutrient intake can alter ${\text{NO}}_{2}^{-}$ content through increases in NO synthase (NOS) and it is involved with decreased heart contraction rate [32]. There was a similar response in salivary ${\text{NO}}_{2}^{-}$ content for both athletes in this study, although there were notable differences in water and carbohydrate intake during RWL between Athlete 1, without diet accomplishment and Athlete 2, with nutritional accomplishment.

Therefore, ${\text{NO}}_{2}^{-}$ content does not appear to be associated with a better recovery of upper limb strength in Athlete 2 when compared with Athlete 1. The differences in nutrient and water intake observed between athletes could partially explain these results by other non-measured mechanisms involving muscular glycogen content and sodium concentration, possible altered by RWL.

Additionally, the effect of RWL on isometric handgrip strength was interesting and controversial. It has been demonstrated that isometric leg strength was decreased in dehydrated state as compared with a hydrated condition [33]. However, the strategy used for dehydration on the mentioned study included jogging for 20 min, which may probably decrease performance, independent of the hydration state. Similarly, the results from the present study revealed that the MMA athletes evaluated did not present notable decreases in handgrip strength following RWL.

## 5. Conclusion

In conclusion, the findings of this study indicate that rapid weight loss resulted in negative effects on salivary nitrite, muscle and cognitive performance in the athletes evaluated. On the other hand, 36 h seems to be sufficient to recover these parameters when the amount of RWL is 5.3% of total bodyweight, while this is not true for RWL of 9.1% bodyweight. Original studies with more subjects are necessary to understand the effects of RWL strategies and amount of RWL on performance of MMA athletes.
